# Determinants of antiretroviral adherence among HIV positive children and teenagers in rural Tanzania: a mixed methods study

**DOI:** 10.1186/s12879-015-0753-y

**Published:** 2015-01-31

**Authors:** Daniel Nyogea, Sally Mtenga, Lars Henning, Fabian C Franzeck, Tracy R Glass, Emilio Letang, Marcel Tanner, Eveline Geubbels

**Affiliations:** Ifakara Health institute, P.O Box 153, Ifakara, Tanzania; Swiss Tropical and Public Health Institute, Basel, Switzerland; ISGlobal, Barcelona Ctr. Int. Health Res. (CRESIB), Hospital Clínic - Universitat de Barcelona, Barcelona, Spain; University Hospital of Zurich, Zürich, Switzerland

**Keywords:** ART adherence, Children, Teenagers, Pill count, Non-parental caretaker, Focus group discussions, In-depth interviews

## Abstract

**Background:**

Around 3.3 million children worldwide are infected with HIV and 90% of them live in sub-Saharan Africa. Our study aimed to estimate adherence levels and find the determinants, facilitators and barriers of ART adherence among children and teenagers in rural Tanzania.

**Methods:**

We applied a sequential explanatory mixed method design targeting children and teenagers aged 2–19 years residing in Ifakara. We conducted a quantitative cross sectional study followed by a qualitative study combining focus group discussions (FGDs) and in-depth interviews (IDIs). We used pill count to measure adherence and defined optimal adherence as > =80% of pills being taken. We analysed determinants of poor adherence using logistic regression. We held eight FGDs with adolescent boys and girls on ART and with caretakers. We further explored issues emerging in the FGDs in four in-depth interviews with patients and health workers. Qualitative data was analysed using thematic content analysis.

**Results:**

Out of 116 participants available for quantitative analysis, 70% had optimal adherence levels and the average adherence level was 84%. Living with a non-parent caretaker predicted poor adherence status. From the qualitative component, unfavorable school environment, timing of the morning ART dose, treatment longevity, being unaware of HIV status, non-parental (biological) care, preference for traditional medicine (herbs) and forgetfulness were seen to be barriers for optimal adherence.

**Conclusion:**

The study has highlighted specific challenges in ART adherence faced by children and teenagers. Having a biological parent as a caretaker remains a key determinant of adherence among children and teenagers. To achieve optimal adherence, strategies targeting the caretakers, the school environment, and the health system need to be designed.

## Background

At the end of 2012, an estimated 35.3 million [32.2–38.8 million] people were living with HIV. The UNAIDS global report 3.3 million children had HIV globally, 2.9 million in sub-Saharan Africa [1]. In 2012, there were 230,000 children living with HIV and 1.3 million orphaned by AIDS in Tanzania [2]. Treatment of HIV infected patients with ART has resulted in a dramatic reduction in HIV related morbidity and mortality [3]. Successful treatment results in virological suppression, an increase in the CD4+ T cells count, and improvement in the clinical well-being of the individual, manifesting as weight gain and resolution or control of opportunistic infections [4]. Adherence to treatment regimens is a prerequisite for the efficacy and durability of any ART [5,6]. According to recent studies, ART regimens require 70–90% adherence in order to be effective [7]. Some studies show that viral suppression for patients treated with non-nucleoside reverse transcriptase inhibitors (NNRTI) is possible with adherence levels ranging from 54%–100% [8]. Poor adherence to ART regimens results in incomplete suppression of HIV replication and emergence of resistance to ART that increase the potential for treatment failure, compromising future treatment options and leading to increased risk of mortality [9]. The World Health Organization (WHO) recommends regimens involving tablets and that syrup or liquid formulation be prescribed for children depending on weight, however recognizing that lack of refrigeration and the supply chain for syrup or liquid forms may bring some challenges [10]. Children and adolescents with HIV often face other life stressors that affect their ability to achieve optimum adherence, including parental HIV disease, poverty, and limited or inconsistent social support [11]. Availability of adherence information assists health care workers in providing optimal care to patients.

In Tanzania, studies on adherence have been primarily focused on adults and less information is available on children and teenagers. As children form a special group due to lengthy expected time on ART and challenges faced during adolescence, more information is needed in order to design appropriate interventions to improve or maintain sufficient ART adherence levels. This study estimated adherence levels, investigated determinants for ART adherence and explored barriers and facilitators of adherence among children and teenagers in rural Tanzania.

## Methods

### Study design

We used a sequential explanatory mixed methods study design. A quantitative cross sectional study was followed by a qualitative study combining focus group discussions (FGDs) and in-depth interviews (IDIs).

### Study setting

We conducted the study in Ifakara town in the Kilombero district of the Morogoro region for 6 months between November 2011 and April 2012. The study was performed within the observational HIV cohort, Kilombero and Ulanga Antiretroviral Cohort (KIULARCO), at the Chronic Diseases Clinic Ifakara (CDCI) in St Francis Referral Hospital (SFRH). The CDCI started providing HIV care and treatment in 2005 in Ifakara [12-14] in accordance with guidelines of the Tanzanian National AIDS Control Program (NACP) [15]. Follow up visit are scheduled every 3–6 months. All patients were treated as per Tanzania National Guideline for HIV/AIDS treatment [16]. During the study, liquid formulations were only available for prophylaxis among HIV-exposed infants and not as treatment for HIV infected children. Early infant diagnosis (EID) was unavailable at CDCI during the study period but has been recently introduced.

### Quantitative component

#### Study participants

We targeted all patients attending the CDCI who were on ART and aged between 2–19 years. Patients were included if they had visited the CDCI for a drug refill between 10–150 days prior to the start of the study. The study participants were traced and interviewed at home during an unannounced visit. For the quantitative data collection, children below 18 years were interviewed together with their parents/caretakers. We did not interview children who were critically ill.

### Data collection

The interviews were conducted in Swahili, the national language of Tanzania and commonly spoken by Ifakara residents. For the quantitative study, data were collected by trained non-medical field interviewers using a structured questionnaire.

### Variables

#### Outcome

The primary outcome variable was ART adherence. Percentage adherence was measured as the ratio of drugs actually taken to the drugs supposed to be taken (prescribed) by using the formula [Adherence = (Number of pills dispensed - Number of pills remained) × 100)/ (Number of pills prescribed per day × number of days between pharmacy visit and home visit)]. The drugs actually taken were estimated by calculating the number of dispensed drugs minus the drugs found during the unannounced visit. This was assessed using pill count, which was first calculated by field worker and then verified by a field manager. The overall average adherence was calculated in two steps. Since commonly used ART regimens involve a combination of three drugs and are dispensed as either a fixed dose or a separate combination, the adherence percentage level for each drug was calculated. Then the average adherence for each regimen was assessed for each individual. Participants were encouraged to provide all ART pills that were dispensed at the clinic in the last visit plus the ones that remained from the prior visit. For individuals whose adherence levels exceeded 100%, the adherence level was capped at 100%. For the analysis, optimal adherence was defined as > =80%.

#### Predictors of ART adherence

The determinants for ART adherence were : gender, pill burden, visit to a local healer (who claimed to have a cure for HIV/AIDS), disease progression measured by age-specific immunological criteria (immune-suppressed if CD4 < 500 cells/mm^3^ for children aged <6 years and if CD4 < 350 cells/mm^3^ for children ≥ 6 years) and WHO clinical staging (I/II versus III/IV), child awareness of HIV disease, parental status (both parents, single parent, or non-parental caretaker (non-parental caretaker included patients grandmother, sister, aunt, uncle or step mother)), distance to the clinic, duration under ART, knowledge about ART treatment duration, HIV support group membership, adherence assistance (parents versus others), socioeconomic status (SES) and education level. Education level was assessed by combining age and school attendance (preschool age (2–5 years), children of school age (6 years or more) who never went to school, children of school age and in school, children of secondary school age (12 years or more) but in primary school, and children of secondary school age and in secondary school).

#### Collection of socioeconomic status (SES) data

Data on socioeconomic status was collected using an asset survey from June 2011 to September 2011 among 1935 patients under ART enrolled in KIULARCO. ‘Low’, ‘middle’ and ‘high’ tertiles of socio-economic status were constructed using principal component analysis [17] on scores calculated from asset ownership (electricity, lamp, radio, television, mobile phone, land line, iron, refrigerator, wrist watch, bicycle, motor bike, motorcar, and having a bank account) and the house’ building materials (mud, bamboo, wood, tiles, cement, carpet, grass, post, brick (sun), brick (fire), wood, cement bricks, iron sheets, tiles, concrete and fabricated bricks).

### Bias

Classification bias of adherence level was minimized by training field interviewers on how to assess and calculate adherence percentages as well as cross-checking adherence estimates for confirmation by a field manager before data entry. To reduce social desirability bias, information on level and determinants of adherence was collected by non-medical staff.

### Sample size

We included all active patients aged 2–19 years under ART attending the CDCI within the last 150 days, i.e. 163 patients. Using an alpha of 5%, a power of 90% and a hypothesized adherence prevalence of 70% in the reference category, with this effective sample size we were able to statistically significantly detect a risk ratio of 1.4.

### Data processing and analysis

Quantitative data were double entered using EPI-DATA (EpiData Association, Odense, Denmark) and analyzed using STATA 11.0 (STATA Corp., College Station, Texas, USA). Chi square test was used to assess the association between the variables studied and the response status and with the adherence categories. Logistic regression was used to estimate associations between adherence and risk factors. Participants with missing adherence (outcome) data were excluded from analysis. Sensitivity analyses were done to assess the potential influence of missing values for predictor variables. Variables with a P-value of less than 0.25 were eligible for inclusion in the multivariable analysis. Since this applied to only one variable (parental status), we reported only the univariate analysis. We assessed if non-response was associated with variables under the study. Variable(s) that were found to predict adherence were investigated in the qualitative part to understand the mechanism underling such association.

### Qualitative component

#### Population and sample

We targeted adolescents aged 13–17 years who were on ART who were aware of their HIV status and the parent or non-parent caretakers in charge of the child. The Tanzanian definition of adolescents has been adopted from the WHO [18] which defines adolescent as a young person aged between 10–19 years. However, for the purpose of this study, we interviewed adolescents of age 13 to 17 years assuming that these adolescents will be capable of expressing their views on issues related to treatment experience and challenges. From the CDCI database we randomly sampled boys and girls aged 13–15 and 16–17 and within each group conducted one FGD. In total we included 35 HIV infected adolescents in the FGDs. We also conducted FGDs with 21 parents or caretakers living with HIV infected adolescents (on ART) residing within the study site, two FGDs with women and two with men. Each FGD comprised of 7–8 participants. We opted for the FGDs due to their usefulness in enhancing social interaction different from other qualitative methods [19], high face validity [20] and their relevance in providing opportunity to interview several participants systematically and simultaneously [21]. To deepen our understanding of issues emanating from FGDs, we subsequently conducted four IDIs, with a 16 year old male patient, a 43 year old male caretaker of an eight year old child and with two healthcare providers (a medical doctor and a nurse).

### Data collection

An interview topic guide with specific themes aided the FGDs. The was guide composed of broader themes that relate to attitude and perception on ART, perceived benefit of ART treatment, perceived risk of treatment, knowledge about treatment adherence, family strategies to ensure treatment adherence, children’s experience with treatment and perceived barriers to treatment adherence. Interview topics were drawn from the relevant literature [22], health behavior theories [23,24] and life experience/observation. The second author who is a female social scientist facilitated the discussions aided by a trained male research assistant. To enhance freedom of expression during FGDs, participants’ were facilitated by researchers of matching sex and in a neutral venue, i.e. at a school after school hours. The discussions were audio recorded following permission from the participants. Throughout the interview, free flowing discussion was encouraged and some new topics which were raised in the first interview were again probed in the next interviews following the principles of grounded theory [23]. Unlike adults, children’s explanations about barriers to treatment adherence were in short segments, sometimes not very direct but useful. The researcher took time to probe and motivate children to provide their inside views and experience that relates to taking the lifelong treatment.

### Data analysis

After the interviews, the research assistants transcribed verbatim digitally audio recorded interviews in Swahili language. The second author frequently reviewed the transcripts using the side notes and relevant ideas were noted. Thematic content analysis was used to process all participants’ descriptions along with identification of relevant concepts and ideas found in the transcripts linked to the topics of inquiry [25].

Relevant ideas were categorized under specific themes and later coded [26]. We included the pre-existing themes, for example “attitude towards ART treatment” and “non-parental care”. We also included emerged themes such as “supportive school environment” and “unfriendly nature of counseling services”.

### Ethical statement

The study received ethical clearance from National Institute of Medical Research of Tanzania. Parents and caretakers signed the informed consent to participate in the study, both for themselves and on their children’s behalf. Participating children assented to answer questions. Records were anonymized and patients were identified by unique identifiers during analysis and reporting. As per routine procedures in the clinic, patients who had poor adherence levels were further followed up by physicians in the CDCI.

## Results

### Data flow

**Figure 1:**
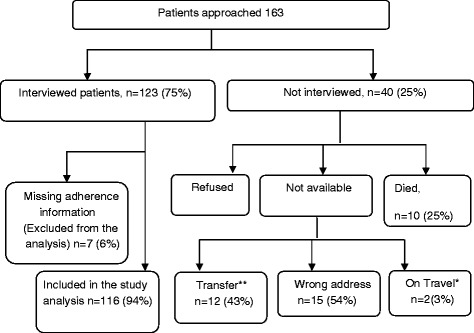
**Flow diagram of study participants of quantitative component.** *On Travel meant the patients was not within Ifakara town. Subjects had traveled outside Ifakara for various reasons. **These are patients who transferred out without having transfer permit from the clinic.

There were 265 HIV patients <20 years of age attending the clinic of whom 163 (62%) were currently on ART (Figure 1). Forty patients (25%) were not interviewed because of refusal, non-availability or death. The reasons for non-availability were undocumented transfer, an incorrect home address or travel of the patient. For the quantitative study, we interviewed 123 (75%) of 163 patients approached. Among the patients successfully interviewed, seven were excluded from the analysis because they did not have adherence data (they did not provide their pills during the home visit). We finally included 116 children and adolescents in the analysis (Figure 1). Non-response was not associated with the variables under study (Table 1).

**Table 1:** **Summary of characteristics of patients by response status**

	**Responders**		**Non-responders**		**P-Value**
**Variable**	**n**	**%**	**n**	**%**	
**Sex**					
Male	67	58	24	51	
Female	49	42	23	49	0.40
**Age categories (yrs)**					
2-5	30	26	16	34	
6-11	46	40	15	32	
12+	40	34	16	34	0.51
**WHO stage**					
Stage I & II	31	27	14	30	
Stage III & IV	40	34	27	57	
Missing	45	39	6	13	0.59
**Distance to clinic**					
<5 km	93	80	43	91	
>5 km	23	20	4	9	0.08
**Duration on ART (yrs)**					
0-2	46	40	11	23	
>3	60	52	20	43	
Missing	10	9	16	34	0.43

WHO stage = World Health Organization clinical stage; yrs = years; km = Kilometers; % = Column percentages.

### Study participants characteristics

Sixty-seven (58%) of the participants were males. The median age was 9.8 years (interquartile range (IQR): 5.7 – 13.3, range 2–19), and 77 (66%) of the participants were not living with both parents (out of these, 61% (47/77) were not living with either parent). Patients had been taking ART for a median of 35.8 months (IQR: 23–46) (Table 2). All participants were taking first line treatment regimens based on 2 NRTIs + 1 NNRTI.

**Table 2:** **Summary of characteristics of participants by adherence categories**

	**Optimal**		**Suboptimal**		**P-Value**
**Variables**	**n**	**%**	**n**	**%**	
**Sex**					
Male	46	69	21	31	
Female	35	71	14	29	0.75
**Age-school**					
Pre-school age 2-5 yrs	23	77	7	23	
>6 yrs never been to school	11	58	8	42	
Primary school age and in primary	21	70	9	30	
Secondary school but in primary	19	70	8	30	
Secondary school age and in secondary	7	70	3	30	0.74
**Baseline CD4 + cell count**					
Below 350 cells/mm3	33	66	17	34	
Above 350cells/mm3	42	71	17	29	
Missing	6	86	1	14	0.54
**Age specific immunosuppression**					
Not Immunosuppressed	61	70	26	30	
Immunosuppressed	16	67	8	33	
Missing	4	80	1	20	0.83
**Duration under ART**					
0-2 yrs	30	65	16	35	
>2 yrs	41	68	19	32	
Missing	10	100	0	0	0.09
**Parental status**					
Both parent	32	82	7	18	
Single parent	20	67	10	33	
Non-parental caretaker	29	62	18	38	0.11
**Knows that treatment is lifelong**					
Yes	57	70	24	30	
No	24	69	11	31	0.85
**Disclosure of HIV status**					
Yes	25	64	14	36	
No	53	75	18	25	
Missing	3	50	3	50	0.29
**Adherence assistance**					
Parents	48	74	17	26	
Non-parental adherence assistants	33	65	18	35	0.29
Visited traditional healer for HIV cure*					
Yes	15	75	5	25	
No	66	69	29	31	
Missing	0	0	1	100	0.28
**Pill burden**					
1 pill	35	69	16	31	
2-3 pills	46	71	19	29	0.80
**Joined any HIV support group**					
Yes	12	57	9	43	
No	69	73	26	27	0.16
**WHO clinical staging**					
Stage I & II	20	65	11	35	
Stage III & IV	26	65	14	35	
Missing	35	78	10	22	0.33
**Socioeconomic status**					
Low	30	73	11	27	
Median	16	73	6	27	
High	27	63	16	37	
Missing	8	80	2	20	0.61

Poor adherence = less than 80%;optimal adherence = 80% or more; WHO = World Health Organization;yrs = years; * visited a healer who claimed to have a cure for HIV/AIDS; Immune-suppressed if CD4 < 500 cells/mm^3^ for children aged <6 years and if CD4 < 350 cells/mm^3^ for children aged 6 years and above;% = Row percentages; p = Chi2 P-value.

### Adherence levels

The overall average ART adherence level was 84.2% with a range from 2.3% to 100% and the proportion of patients with adherence level of 80% or more was 70% (81/116).

### Determinants for adherence

From the logistic regression analysis, children living with non-parent caretakers were more likely to have poor adherence compared to children living with their parents ((OR = 2.84, 95% CI: (1.04-7.77)) (Table 3). Children living with single parent or non-parent caretaker had lower levels of optimal adherence compared to children living with biological parents. The magnitude of association between adherence and educational status was high, but the evidence of association was statistically non-significant (children of primary school age (6 years or more) who never went to school compared to preschool age children, OR = 2.39, 95% CI: (0.69-8.28)). Children who reportedly joined an HIV support group had a lower proportion of optimal adherers compared to children who were never members (OR =2.0, 95% CI 0.75-5.27). The quantitative analysis showed no associations between adherence levels and sex, education, duration on ART, CD4+ T cells count, WHO clinical staging, SES, ever visited traditional healer, pill burden and disclosure status.

**Table 3:** **Univariate logistic models of poor ART adherence among children and teenagers in Ifakara, Tanzania (n = 116)**

	**Univariate analysis**		
**Variable**	**OR**	**95% CI**	**P-Value**
**Sex**			
Female	0.88	0.39-1.96	0.748
**Age-School**			
Pre-school age 2-5 yrs	1		
>6 yrs never been to school	2.39	0.69-8.28	0.170
Primary school age and in primary	1.41	0.45-4.45	0.560
Secondary school age but in primary	1.38	0.42-4.5	0.591
Secondary school age and in secondary	1.41	0.29-6.9	0.674
**Baseline CD4 + cell count**			
Below 350 cells/mm3	1		
Above 350 cells/mm3	0.79	0.35-1.77	0.561
**Age specific immunosuppression**			
Not Immunosuppressed	1		
Immunosuppressed	1.17	0.46-3.08	0.746
**Duration under ART**			
0-2 years	1		
>2 years	0.88	0.38-1.96	0.735
**WHO stage**			
Stage I & II	1		
Stage III & IV	0.98	0.37-2.61	0.966
Visited Traditional Healer for HIV cure*			
No	1		
Yes	0.76	0.25-2.28	0.623
**Pill burden**			
1 pill	1		
2-3 pills	0.90	0.41-2.0	0.803
**Disclosure of HIV status**			
Yes	1		
No	0.61	0.26-1.41	0.246
**Joined any HIV support group**			
No	1		
Yes	2.0	0.75-5.27	0.166
**Adherence assistance**			
Parents	1		
Non-parental adherence assistants	1.54	0.69-3.41	0.288
**Knows that treatment is lifelong**			
**No**	1		
**Yes**	1.65	0.71-3.84	0.246
**Socio-economic status**			
Low	1		
Median	1.02	0.32-3.3	0.970
High	1.62	0.64-4.08	0.310
**Parental status**			
Both Parents	1		
Single parent	2.29	0.75-6.98	0.200
Non-parental caretaker	2.84	1.04-7.77	0.042

*Visited a healer who claimed to have a cure for HIV/AIDS; Poor adherence = less than 80%;optimal adherence = 80% or more; Immune-suppressed if CD4 < 500 for children aged <6 years and if CD4 < 350 for children aged 6 years and above; WHO = World Health Organization; yrs = years; CI = confidence interval; OR = odds ratio.

### Barriers and facilitators of adherence

The results of the FGDs and IDs complemented the cross-sectional survey and shed further light on its findings. The thematic analysis suggested a range of themes influencing adherence to ART, many of which were commonly shared among the FGD and IDIs participants. The summary of the barriers and facilitators of ART adherence are found in Table 4.

**Table 4:** **Barriers to treatment adherence from the qualitative component of the study–as derived from focus group discussions and in-depth interviews**

**Category**	**Treatment adherence barriers**
**Stigma related factors**	▪ Fear of disclosure of child’s status to the teachers and family members by parents
	▪ Segregation by teachers and other children
**Lack of supportive environment at home**	▪ Inadequate support from parents
	➢ Parents move to farming areas without one to follow up on child’s treatment schedule
	➢ Men do not provide enough treatment adherence support
	➢ Extended family members do not provide support
**Lack of supportive environment from school**	▪ Teachers do not provide adequate support to children on ART
**Health service delivery factors**	▪ Lack of child friendly counseling services
	▪ Inconvenient treatment schedule
	▪ Longevity of treatment
**Patient factor**	▪ Forgetfulness
	▪ Feeling better

### Facilitators of treatment adherence

#### Positive attitudes and perceived benefits towards treatment

Both children and caretakers reflected a positive attitude towards treatment. This was indicated by the adolescent’s expression of *“feeling good with the treatment”* which was a common statement by most of the children. Caretakers shared the same experience “*we become happy when we see health improvement of our children*”.

Positive attitude towards treatment was also reflected by the experiences reported by the groups in relation to the perceived benefits of treatment. The most recurrent themes related to the perceived benefits of treatment included the *‘reduction of recurrent diseases’, “looking healthy after the initiating ARV”, “living longer”, “getting cured”, “ability to participate in economic activities” and “ability to attend classes”.*

The following excerpts indicate some of the related themes:

*“My son used to be sick every day, and he was very thin, but after treatment initiation I can see that he is now well, but I can see other children also, before they start treatment they become very sick but after treatment they change (their health status).* [FGD, Female].

*“Ooh, the treatment is good, like me I wasn’t like this, before taking this treatment I had rashes all over my body and I was so thin until my fellow children were avoiding me and said she has AIDS, but when I started taking the medicine, my condition changed and I am now big and healthy”.* [FGD, Girl].

### Knowledge about treatment adherence

Generally, participants were able to explain the meaning of treatment adherence conditions. The most frequently mentioned treatment adherence conditions included *“taking drugs in the morning and evening”*, *“taking drugs without stopping”, “taking drugs before food”, “eating fruits”* and *“abiding to the time of taking drugs without changing”.*

For those who were able to mention the risks of non-adherence to treatment, they mostly mentioned recurrence of illness, suffering and death as reflected in the following excerpts:

*“Following treatment instructions it means* t*aking drugs every day because at times the body is used to that. When you stop abruptly the body will get problems because it will notice the difference. And the body will get fevers which were over”.* [FGD, Female].

### Treatment adherence barriers

Despite respondents clearly valuing ART, several barriers existed for maintaining ART adherence in the social environment, in the health service delivery, and related to the treatment and disease itself.

### Fear of stigma and consequences

Most caretakers and children were strongly opposed to sharing the child’s treatment status with other people, friends or relatives in the community due to fear of mocking by other people in the community. Most children and adolescents viewed taking their treatment pills in front of their fellow children as unacceptable due to negative reaction from other children in the community:

*“I can’t dare to tell any of my friends or neighbors that my child is on treatment, because they will start spreading this information to others*”. [FGD, Male].

*“When I take these drugs they (other children) will say, “We do not know what he is suffering from. He is taking drugs every day; but he is not getting cured”.* [FGD, Boy].

Enacted stigma also contributed to a lack of a supportive school environment. The school mates and teachers mocked, segregated and stigmatized children with HIV taking ART. Some children reported that they initially used to take their drugs at school but have decided to change and begin to take drugs at home due to some insults from their fellow students.

*“Parents fear disclosing their children’s HIV and treatment status to teachers because of fear that their children may be segregated. My child was pinned a red label on his shirt by his teacher after he knew the HIV and treatment status of the child”.* [FGD, Male].

*“In the past fellow schoolmates started mocking at me that I have AIDS after they saw me taking ARV at school. Later I had to change and started taking the pills at home”.* [FGD, Girl].

### Non-parent caretaker

According to the FGD discussants, children who live with their biological parents receive much more treatment support and care as compared with children who live with their caretakers.

“*Step mothers contribute also to poor adherence. At times it happens that you are sick and the father asks the mother to take you to the hospital. When dad goes to work, the step mother doesn’t take me for treatment and I continue suffering*”. [FGD, Boy].

### Health service delivery factors

Some children and caretakers expressed their concern about the counseling sessions offered at the health facility which combined adults and children all together. Children felt that they need to have separate counseling sessions so they can express their treatment challenges freely without having adults interfering and can receive the attention they need from the counselors.

*“They combine us (children) with those (adults), when we are there with them (adults) we cannot understand things (information) and some of us cannot ask questions or explain problems…. There should be some separate sessions for children and so the doctors could listen nicely to children”.* [FGD, Boy].

### Not informed about HIV status

Some children expressed their concern that they were not informed about their HIV status by their parents. One child described his concern as follows:

*“I grew up taking medicine without knowing the disease that I was suffering. They kept on telling me that I have malaria and until now they have not told me anything with regards to HIV, but the doctor told me that I am HIV positive and I feel bad that my sister did not tell me”.* [FGD, Boy].

Some parents also explained difficulties faced in disclosing HIV status to their children but once informed the children were able to adhere with their treatment schedule, as explained by one parent in the following excerpt:

*“I asked my child to take medicine without telling him what he was suffering from. When I told him what he was suffering from (HIV), it helped him to follow the instructions. I told him that his mother died because of the same problem and that he should not stop taking drugs”.* [FGD, Male].

### Preference of traditional medicine

Some parents encourage their children to go beyond the formal health sector and take traditional medicine instead of drugs obtained at the health facility.

*“Some parents do not want their children to take medicines from the hospital. They give them traditional medicines when it is obvious that the child has AIDS”.* [FGD, Boy].

### Inconvenient treatment schedule and longevity of the treatment (treatment fatigue)

Taking drugs in the morning before going to school was not very patient-friendly as children often forget while hurrying for school or felt sick if they were forced to take drugs before breakfast. One boy expressed the concern as follows:

“*My mother tells me to take drugs in the morning and I sometimes wake up very early and no food prepared, and when I take medicine (in the morning) I feel nausea but I go to school just like that… and I try not to miss school*…”. [FGD, Boy].

Children and caretakers gave the impression that some children are getting tired of taking the medications as explained below:

“*Some grown-up children may sometimes cheat that they have taken drugs while they have not, because they are tired and the parents believe that they have already taken the drugs”.* [FGD, Female].

*“Some children are tired of taking medicine, I remember one day when I was giving my child medicine, and he told me that, ‘uncle if you want to give me medicine give me even those for tomorrow’”.* [FGD, Male].

### Forgetfulness and feeling better

Patients FGD discussants from both groups mentioned the problem of forgetting to take drugs at the appropriate time due to the intensity of their engagement in games. At times, especially when patients observe some health improvement, they do not have the motivation to continue with treatment.

*“There are others (children) who put priority on games and when they are playing they forget to take drugs completely”.* [FGD, Boy].

*“You find another person goes to the hospital and when they are given drugs they take them for one month and in the next month they stop taking these drugs when they see that they are OK”.* [FGD, Girl].

## Discussion

This explanatory mixed-methods study among children and teenagers attending a large HIV care and treatment clinic showed that participants had a positive attitude towards ART and 70% of the participants achieved desired level of adherence (defined as 80% or higher). The proportion of patients with acceptable levels of adherence varies across several studies using different techniques of evaluating adherence.

A review of ART adherence in low and middle-income countries found a range in adherence level estimates from 49% to 100% with 76% of articles reporting >75% adherence [27]. A study done in India showed 95.3% [28] of the children had acceptable level of adherence whilst a study done in Dar es Salaam showed 97% [29] of children had optimal adherence. Another study done in Ethiopia showed that 34.8% of the children had acceptable level of adherence when unannounced home visit was made [30]. The wide difference here appears to be driven by the nature of the visits (announced versus unannounced or prior known visit). Possible explanations for a low adherence in our study might be: (a) by interviewing participants at home, we might have captured children who were at risk of being “lost to follow-up”; in clinic-based studies these children would not have been included; (b) we used non-medical staff to collect information which may have reduced social desirability bias; (c) measuring adherence by pill count usually shows low adherence levels compared to other methods such as self-report [31] and lastly (d) our study participants had an average treatment duration of 3 years, and as the qualitative part outline treatment fatigue was present.

Patients not staying with biological parents were more likely to have poor adherence. Other studies found that children’s adherence is affected by their dependence on caretakers and that if adult caregivers are unavailable, the risk of missed doses increases [32,33]. The successful treatment of a child requires the commitment and involvement of a responsible caregiver yet qualitative findings showed that most often both parents and children are concerned with the stigma related to disclosure of HIV status to other family members or caretakers, friends and schools teachers thus restricting the child’s options for seeking support [10]. According to our participants, the care given to HIV infected children by biological parents was not the same as that given by non-parental caretakers. This may be partly because some non-parental caretakers were unaware of the child’s HIV status. In other studies, disclosure was related to improved adherence to ART medications and influenced children’s participation in healthcare decision-making [34-37]. Health illiteracy might be another issue surrounding poor adherence levels. Compared to adults, children have lower levels of health literacy and thus, following health care providers’directives on maintaining adherence might not be easy for them [32]. In times when children focus too much on playing with friends, they tend to forget taking the drugs and the situation worsens if their caretakers are not present. Children who live with HIV-positive parents have the potential additional benefit of the parental experience in living with HIV and taking ART. However, we did not have information on the HIV status of parents or caretakers. Children with non-parental caretakers were older (median age 11.4 versus 8.3 years) suggesting that some adherence problems might be partially attributed to puberty. However these possible explanations should be investigated further.

Secondary school aged children who had only primary education or were still in primary school were more likely to be non-adherent, although the association was not significant. This may be partly explained by the fact that 50% of these children were living with non-parental caretakers, thus were at risk of less support to maintain adequate adherence levels. The study’s qualitative part provides another explanation for these findings. The morning dose was challenging for children attending school as they often have to leave for school early in the morning before breakfast is ready. Competing demand between taking the morning ART dose and rushing to school make them vulnerable to skip their medications. A study on how food insecurity impacts on non-adherence to ART found that side effects were exacerbated when taking ART in the absence of food [38]. However, our study’s participants reside in a location where food supply is quite stable and food shortage was not reported as a barrier for optimal adherence in the qualitative component. It was rather the school environment where HIV patients experienced mocking and segregation which made it hard for them to take their ART while at school and led to missing the morning dose. In some settings HIV positive children attending school were given names by their colleagues so as to victimize them. In Namibia for instance, it was found that derogatory terms have been used to discriminate HIV students [39]. This impacts adherence levels as low social support [40] and stigma has been identified in previous studies to affect access to health care, social interaction and medication adherence [41].

The association between adherence and SES, although not significant, is in an unexpected direction; patients coming from affluent families are at higher risk of poor adherence. The effect of SES on adherence among HIV infected patients is considered a controversial issue [42-44]. Suggested pathways in which socioeconomic status might be associated with adherence include education's effect on shaping a financially stable future, and on acquiring health literacy and knowledge to use health resources, while income plays a big part in obtaining better housing conditions, recreational facilities and better health care [45]. However, a systematic review of the evidence regarding the association of SES with adherence to treatment of patients with HIV/AIDS found no conclusive support for existence of a clear association between the two variables [46]. From the explanatory qualitative study, barriers of optimal adherence were unfavorable school environment, patients being unaware of their own HIV status, children feeling too shy to go to collect medication at the clinic, parents’ failure to disclose the children’s HIV status to family members/caretakers and differential care from non-parental caretakers.

### Strengths and limitations of the study

Apart from the strengths aforementioned, using a mixed methods design allowed explanation and in-depth understanding of the quantitative results. The patients were set in programmatic conditions i.e. not in a clinical trial cohort with intensive support. The study covered a wide time window, thus increasing generalizability and reducing potential seasonality bias.

In addition to limitations of the methods used for measuring adherence, selection bias may have resulted from the rather high non-response rate of 25%. However non-response analysis showed that variables under study were not associated with non-response. In addition, we performed sensitivity analysis (data not shown) for predictor variables with missing information and the results were not considerably affected except WHO staging. However WHO staging was taken from the clinic database (and not the home visit) and was often missing due to drug refill visits by someone other than the patient. In some studies, pill count has been found to predict response to ART [47], particularly when conducted with no prior notification. However, in other studies, it has been shown to be liable to pill dumping [48], fabrication, and manipulation. Grouping a cohort of 2–19 year olds together might have disguised specific adherence challenges for younger children (palatability, caregiver availability), which are very different from the challenges for teenagers.

### Implications

The implications of poor adherence for these children might be that they experience worse clinical outcomes. If this situation extends beyond our immediate setting, both the current and future treatment options are under threat if appropriate actions are not put in to place to address adherence issues in children and teenagers. A systematic review showed that almost a quarter of patients fail second-line therapy within 12 months, mainly because of poor adherence rather than drug resistance [49]. As this age group (12 + yrs) is becoming sexually active, the likelihood of transmission of HIV increases when the drugs are not taken properly and the virus is not suppressed [50]. New efforts have to be made and fully implemented to boost adherence to ART in this important population.

The following recommendations could help improve adherence among children and teenagers in settings similar to ours: Parents and/or caretakers should counsel and guide children on taking drugs parallel with providing a supportive environment at home i.e. family supporter to ensure that during the parent’s absent children are able to maintain good adherence. Caretakers should create conducive environment at home for children i.e. make sure that food is ready before the child take his/her dose especially in the morning. Counsellors should improve counselling techniques and focus more on how to overcome challenges that make children not take their drugs as required. Furthermore, children should have age-specific counseling services at HIV clinics separate from adults. This can be done at the HIV clinic or at home during home-based care (HBC) visit. Assigned caretakers (parents or non-parents) should disclose their children’s HIV and ART status to household members who will be able to provide support to children when parents are absent. Health promotion messages should be tailored to specific groups such as teachers, fellow students as well as general community members to reduce their stigmatizing behavior towards HIV positive children. Advocacy for the formulation of peer support groups in the community and in schools should be encouraged to take a lead in advocating for love, care and support for HIV infected children.

## Conclusion

In conclusion, evidence from this study suggests that, children not living with both parents, are more likely to have worse adherence to ART, in part due to an inadequate support environment in school and within the nuclear or extended family.

## Abbreviation

HIV: Human immunodeficiency virus
ARVs: Antiretrovirals
ART: Antiretrovirals treatment
EID: Early Infant Diagnosis
CDCI: Chronic disease clinic
SFRH: Saint Francis referral Hospital
NACP: Tanzania National Aids control Programme
HBC: Home based care
WHO: World Health Organization
FGD: Focus group discussions
IDI: In-depth Interviews
SES: Socioeconomic status
